# Association of Climatic Factors with Frequency of Dengue

**DOI:** 10.3390/idr18030047

**Published:** 2026-05-16

**Authors:** Gracia Viviana González-Enríquez, Blanca Miriam Torres-Mendoza, Martha Escoto-Delgadillo, Efrain Chavarria-Avila, Sagrario Karina Esparza-Avila, Clara Esperanza Santacruz-Tinoco, Bernardo Martínez-Miguel, Magally Farah Diva Arenas-Sevilla, David Israel Javalera Castro

**Affiliations:** 1 Laboratorio de Apoyo, Vigilancia e Investigación Epidemiológica, División de Neurociencias, Centro de Investigación Biomédica de Occidente, Instituto Mexicano del Seguro Social, Guadalajara 44340, Mexico; gracia.gonzalez@academicos.udg.mx (G.V.G.-E.); sagrario.esparza@imss.gob.mx (S.K.E.-A.); magally.arenas@imss.gob.mx (M.F.D.A.-S.); 2 Departamento de Disciplinas Filosófico, Metodológicas e Instrumentales, Centro Universitario de Ciencias de la Salud, Universidad de Guadalajara, Guadalajara 44340, Mexico; efrazan@gmail.com; 3 Centro Universitario de Ciencias Biológicas y Agropecuarias, Universidad de Guadalajara, Guadalajara 45200, Mexico; martha.escotod@gmail.com; 4 División de Laboratorios Especializados, Instituto Mexicano del Seguro Social, México City 07760, Mexico; clara.santacruz@imss.gob.mx (C.E.S.-T.); bernardo.martinezm@imss.gob.mx (B.M.-M.); 5 Doctorado en Salud Pública, Centro Universitario de Ciencias de la Salud, Universidad de Guadalajara, Guadalajara 44340, Mexico; 6 Departamento de Fundamentos Biológicos, Instituto de Ciencias Biológicas, Universidad Autónoma de Guadalajara, Guadalajara 45129, Mexico

**Keywords:** arboviruses, dengue virus, climate change, temperature, vector-borne, serotype

## Abstract

Background: Climate change has contributed to the global resurgence of dengue, with a spike of more than 14.4 million dengue cases. This study aimed to analyze the association between dengue frequency with climatic factors, circulating serotypes, and disease severity in northwestern Mexico. Methods: A retrospective time-series study was conducted using dengue molecular diagnostic data reported between September 2017 and January 2025 by the Laboratorio de Apoyo a la Vigilancia e Investigación Epidemiológica del Centro de Investigación Biomédica de Occidente, Mexico. Data included dengue frequency, serotype distribution, and clinical severity across seven states in northwestern Mexico (Colima, Guanajuato, Jalisco, Michoacán, Nayarit, Sinaloa, and Sonora). Meteorological data were obtained from the Automatic Meteorological Stations of the National Water Commission. Associations between dengue frequency and climatic variables were evaluated using linear regression models. Statistical analyses were performed using SPSS v24 and R v3.5. Results: In Jalisco, minimum, mean and maximum temperatures, as well as precipitation, were significant predictors of dengue cases, explaining approximately 21.7% of the variance (adjusted R^2^ = 0.217, *p* < 0.001). In Colima and Michoacán, precipitation showed no predictive value. In Guanajuato, the maximum temperature was excluded from the model (adjusted R^2^ = 0.226). Models for Nayarit, Sinaloa, and Sonora excluded two or more climatic variables, with adjusted R^2^ values of 0.111, 0.151, and 0.049, respectively. Conclusions: Climatic conditions and epidemiological time trends explain a modest proportion of dengue cases in northwestern Mexico, with the strongest association observed in Jalisco. Additional determinants, including vector ecology, host immunity, circulating serotypes, population mobility, and public health interventions, should be considered to better understand dengue dynamics.

## 1. Introduction

Dengue is a mosquito-borne viral disease considered by the World Health Organization as a major public health concern [1]. Over the past decades, dengue incidence has increased worldwide, with the exception of 2021, when reported cases declined during the SARS-CoV-2 pandemic [1,2,3]. Dengue outbreaks occur cyclically every three to five years and are influenced by the distribution, density, and competence to infect and transmit the virus of *Aedes* mosquito vectors [2]. In 2024, 14.4 million dengue cases were reported globally, representing more than double to the previous record 2023. This surge included over 52,711 severe cases and 11,185 deaths [4]. In Mexico alone, 125,160 confirmed cases and 478 deaths were recorded, with the highest burden concentrated in northwestern states [5].

Multiple factors contribute to dengue emergence and spread, including vector distribution, introduction of new viral serotypes, population immunity, health system capacity, human mobility, tourism, and climate change. Climatic variables—particularly temperature, rainfall, and humidity—play a critical role by influencing vector survival, reproduction, and viral replication [6,7].

Climate change is altering the distribution and frequency of dengue fever globally, impacting public health and prevention strategies. There are projections for different climate scenarios across various regions of the world [8] and in Mexico [9]. These projections show geographical variations and significant gaps in information on climatic variables, which can also contribute to increased dengue cases. Therefore, it is essential to develop approximate regional scenarios that serve as a basis for future predictions [8,9].

Accordingly, this study aimed to evaluate the relationship between dengue frequency and climatic factors in northwestern Mexico and to describe the circulating serotypes and disease severity.

## 2. Materials and Methods

### 2.1. Study Design and Data Collection

A retrospective time-series analysis was conducted using dengue molecular diagnostic records from 7 September 2017 to 15 January 2025. Data were obtained from the electronic database of Laboratorio de Apoyo a la Vigilancia e Investigación Epidemiológica del Centro de Investigación Biomédica de Occidente (LAVIE-CIBO), which supports epidemiological surveillance of vector-borne diseases in the region.

### 2.2. Eligibility Criteria

Records were included if samples: (1) were analyzed by RT-qPCR for vector-borne disease diagnosis; (2) were collected within 0–5 days of symptom onset; (3) included an epidemiological investigation; (4) met the operational definition of a probable dengue case; (5) originated from one of seven northwestern Mexican states (Colima, Guanajuato, Jalisco, Michoacán, Nayarit, Sinaloa, or Sonora); and (6) corresponded to Instituto Mexicano del Seguro Social beneficiaries.

### 2.3. Laboratory Diagnosis and Serotyping

Dengue virus detection was performed by LAVIE-CIBO using the TaqMan™ Arbovirus Triplex Kit (ZIKV/DENV/CHIKV) (Applied Biosystems, Waltham, MA, USA). Positive samples were subsequently serotyped using the Dengue Serotyping Real-Time PCR (RT-qPCR) Detection Kit (VIASURE, Zaragoza, Spain), following manufacturer instructions. DENV-positive samples with Ct > 32 were not serotyped and were classified as “Not determined”.

### 2.4. Climatic Data

Northwestern Mexico encompasses approximately 438,005 km^2^ (22% of the national territory) [10] and exhibits predominantly dry and semi-dry climates. Daily meteorological data on precipitation and minimum, mean, and maximum temperatures from January 2017 to January 2025 were obtained from the Automatic Meteorological Stations of the Hydrological Information System of the National Water Commission [11].

### 2.5. Statistical Analysis

Descriptive statistics were used to summarize dengue frequencies, serotypes, severity, and climatic variables. The dengue frequencies were obtained from the relationship between positive dengue cases by RT-qPCR and the total number of cases, with the operational definition of a probable dengue case per 100.

Climatic data were aggregated by epidemiological week (52 weeks per year). Weekly averages were used to construct heatmaps and regression models. Linear regression analyses were conducted separately for each state to evaluate associations between dengue rate and climatic variables. Statistical significance was set at α = 0.05, with 95% confidence intervals. The analyses were performed using statistical packages SPSS v24 (IBM, Armonk, NY, USA) and R v3.5 (The R Foundation, Vienna, Austria).

## 3. Results

### 3.1. Dengue Frequency and Serotype Distribution

Between 2017 and 2025, a total of 28,986 dengue cases were diagnosed across the seven states. Jalisco accounted for nearly half of all cases, whereas Sinaloa reported the lowest number. In recent years, a serotype shift was observed from D1, D2 and finally D3. Notably, 66.3% of cases corresponded to non-severe dengue over the years, and 2024 was the year with the highest cases of dengue with warning signs (*n* = 6151) and severe dengue (*n* = 265). The largest outbreak occurred in 2024, coinciding with a post-pandemic resurgence (Table 1).

The cases of dengue and DENV serotypes in the seven states are shown in Table 2. DENV serotype D3 is described as responsible for the most significant number of diagnoses in all states, and subsequently, the frequency of circulating serotypes was different for each state. Likewise, the patients diagnosed were outpatients, with a ratio of 2.35 to 1 for each hospitalized patient, and only 55 deaths were diagnosed with dengue. Regarding the clinical classification of dengue, the cases were distributed according to their definition as NSD with 19,205 (66.3%) cases, DWS with 8885 (30.7%), SD with 656 (2.3%), and “Not clinically classified” with 240 (0.8%) (Table 2).

Serotype D2 showed the highest proportion of severe dengue cases, followed by D3 and D1 (Table 3).

### 3.2. Climatic Conditions and Dengue Cases

Dengue cases exhibited strong seasonality, peaking during warmer and wetter months, particularly between epidemiological weeks 29 and 50 of 2024. States with higher rates experienced moderate rainfall and temperatures favorable to mosquito survival, whereas states with extreme heat and lower precipitation reported fewer cases.

**Figure 1:**
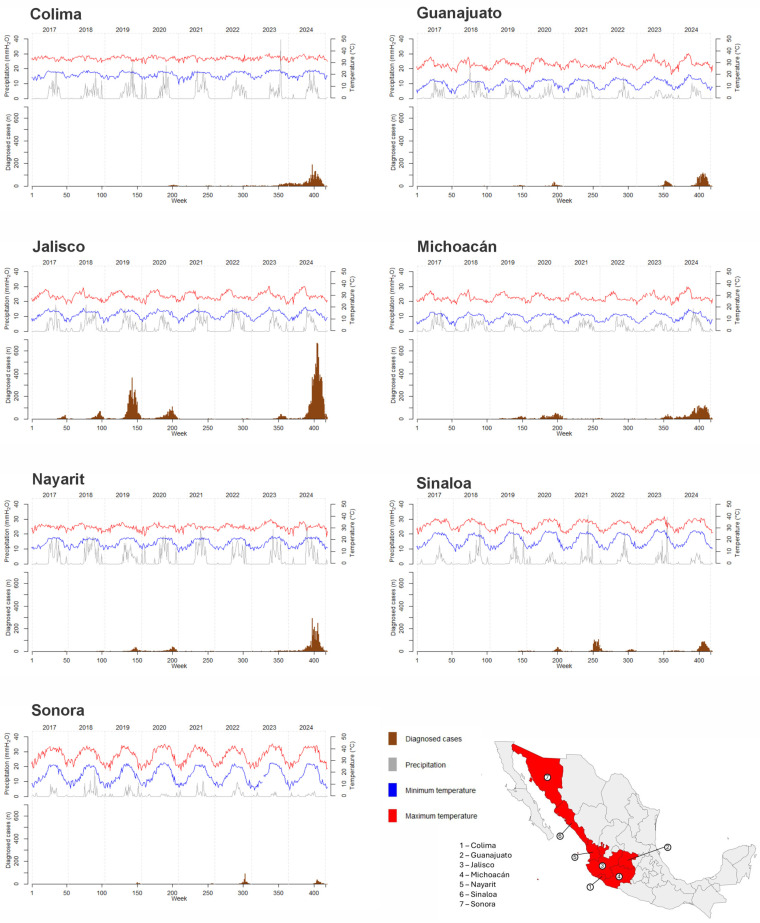
Climatic variables and dengue cases in seven states of northwestern Mexico by epidemiologic week.

Figure 1 indicates the distribution of dengue cases, as well as maximum and minimum temperatures and precipitation, from 2017 to 2025 by epidemiological week (EpiWeek) across the seven states of northwestern Mexico. The highest mean maximum temperatures were recorded in Sinaloa (32.53 °C; range: 24.43–39.76 °C), Colima (33.70 °C; range: 28.86–37.72 °C), and Sonora (34.21 °C; range: 21.10–44.17 °C). The states with the lowest mean minimum temperatures were Michoacán (11.23 °C; range: 3.78–18.21 °C), Guanajuato (12.20 °C; range: 3.26–19.63 °C), and Jalisco (13.96 °C; range: 5.78–20.13 °C). Average rainfall was highest in Nayarit (3.28 mm; range: 0–26.44 mm), followed by Colima (2.71 mm; range: 0–39.54 mm) and Jalisco (2.61 mm; range: 0–18.45 mm).

**Figure 2:**
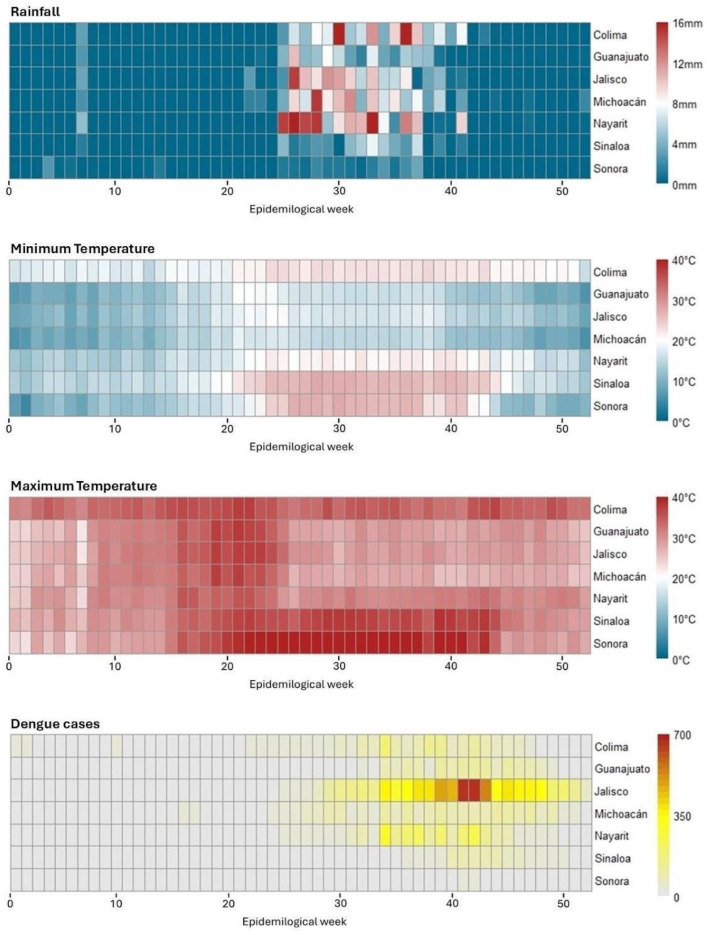
Heatmap of dengue cases in relation to rainfall and maximum and minimum temperatures in the seven states of northwestern Mexico by epidemiological week in 2024.

Across all seven states, dengue cases peaked during the 2024 outbreak, with a marked increase between EpiWeeks 29 and 50. During this period, the states with the highest number of dengue cases were Jalisco, Nayarit, Michoacán, and Colima, which experienced maximum temperatures ranging from 22.46 °C to 37.47 °C, minimum temperatures from 5.68 °C to 23.90 °C, and total rainfall between 0 and 26.44 mm. Conversely, Sinaloa and Sonora, which reported fewer cases, exhibited higher maximum temperatures (25.52–42.65 °C), higher minimum temperatures (12.20–27.60 °C), and lower precipitation (0–7.40 mm) (Figure 2).

### 3.3. Dengue Prediction Models

The analysis of dengue behavior in the region of interest was studied following the reports from 7 September 2017 to 15 January 2025. For proper tracking, the weeks included in this period were numbered from 1 January 2017, giving a total of 419 weeks of reporting. A multivariate analysis was performed to define the predictive value of the meteorological parameters on the number of dengue cases because the rainfall variable has values of zero; only with linear regression were valid models obtained. In addition, the meteorological variables showed a wide dispersion among regions, so a model was made for each state. Table 4 shows the prediction models for each of the seven states of the northwestern region.

The diagnosis of dengue cases in Jalisco decreased during the SARS-CoV-2 pandemic. This unexpected trend complicated the development of an accurate mathematical model for predicting dengue rates. Consequently, tests were conducted for each state to determine which climatological parameters best explained the observed patterns (Table 4).

The model of Jalisco included all the meteorological variables as a predictor of dengue cases, which explains approximately 22% of the cases (adjusted R^2^ = 0.217, *p* < 0.001). For the rest of the states, some meteorological variables were not included in the models (Table 4).

According to the estimates presented, the behavior of dengue is explained using the linear model of the following formula and its substitutions according to the values for each state:

*Dengue cases = (A∙W) + (B∙P) + (C∙T_min_) + (D∙T_mean_) + (E∙T_max_) + Cons*

where

*W*: Week

*P*: Precipitation

*T_min_*: Minimum Temperature

*T_mean_*: Mean Temperature

*T_max_*: Maximum Temperature

*Cons*: Constant

*A*,*B*,*C*,*D* and *E*: These are the values of the estimator corresponding to each variable by region, as presented in Table 4.

State-specific linear regression models revealed heterogeneous associations between climatic variables and dengue rates. The Jalisco model included all meteorological parameters and explained approximately 21.7% of dengue variability. In other states, fewer variables contributed significantly, resulting in lower explanatory power.

## 4. Discussion

This study identified a clear association between dengue rates and climatic factors, particularly minimum and maximum temperature and rainfall. An increase in minimum temperature has been associated with a higher number of dengue cases [12,13,14]. Mosquitoes exhibit prolonged flight activity and greater efficiency at temperatures between 15 °C and 27 °C, which increases the likelihood of human–vector contact [12,15]. In the present analysis, minimum temperatures across the evaluated states ranged from 4 °C to 28 °C, encompassing the optimal range for vector activity.

In contrast, higher temperatures were associated with a reduction in dengue cases, although Childs et al. observed a nonlinear relationship between temperature and dengue incidence [16]. When temperatures exceed 30 °C, mosquito survival and feeding behavior tend to decline [17]. Experimental studies have shown that mosquito viral incubation and larval development periods shorten between 15 °C and 30 °C [18], whereas viral replication and dissemination within mosquito salivary glands are disrupted at approximately 32 °C [15]. In this study, maximum temperatures reached up to 40 °C in Sonora and Sinaloa, the two states that reported fewer dengue cases during the 2024 outbreak.

A systematic review reported that the risk of dengue infection increases by approximately 13% for every 1 °C rise in temperature [17]. Similarly, in Jalisco, our findings indicate that a 1 °C increase in minimum temperature was associated with a 17% increase in dengue risk. This temperature sensitivity may partly explain the magnitude of the most recent outbreak in the region, particularly when combined with the observed shift in circulating serotypes [17,19].

Rainfall was another relevant climatic factor and, in this study, demonstrated a negative association with dengue rate. A similar pattern was reported in Kenya, where heavy rainfall was hypothesized to disrupt mosquito breeding habitats or reduce the effectiveness of vector control measures [20]. However, other studies have reported a positive association between rainy periods and increased dengue risk, highlighting the complex and context-dependent role of precipitation in dengue transmission dynamics [13].

When the combined effects of environmental variables were considered, Jalisco—particularly in 2024—exhibited a convergence of conditions consistent with a worst-case transmission scenario: sustained moderate rainfall, maximum temperatures close to 30 °C, and minimum temperatures near 15 °C for more than six consecutive months. Both univariate analyses and regression models suggest that dengue cases are driven less by isolated climatic variables and more by specific combinations of favorable conditions that can overwhelm public health systems. Identifying and quantifying the relative contribution of these factors constitutes one of the principal contributions of this study.

Globally, a decline in dengue rate was observed between 2020 and 2021 [21,22,23,24]. A similar reduction in confirmed dengue cases occurred in northwestern Mexico during this period; however, a sharp resurgence was observed in 2024, particularly in the state of Jalisco. Comparable post-pandemic increases were also reported in Malaysia, Sudan, and Brazil in 2022 relative to 2021 [21,22,23].

Containment measures implemented during the COVID-19 pandemic underscored the role of human mobility in dengue transmission. In Sri Lanka and Brazil, dengue cases declined by 31% and 40%, respectively, during periods of restricted movement [25,26]. These observations support the hypothesis that population mobility is a critical driver of dengue spread in urban settings [22,26]. Additionally, limited access to healthcare services during the pandemic—due to prioritization of COVID-19 control—may have contributed to underreporting of dengue cases [21].

Regarding viral serotypes, a shift in circulating variants was observed in northwestern Mexico, with a transition from predominance of DENV-1 and DENV-2 to DENV-3. In the most recent year, DENV-3 was the dominant serotype, accounting for a substantial proportion of cases. The introduction of new DENV serotypes, genotypes, or lineages can displace endemic strains and, in immunologically naïve populations, trigger large outbreaks and potentially more severe clinical outcomes [19,27]. In contrast to some previous reports [27,28], DENV-3 was not associated with increased disease severity in this study. Most severe cases were linked to DENV-2, which has been consistently associated with more severe dengue manifestations [28,29]. Both DENV-1 and DENV-2 were associated with thrombocytopenia and bleeding complications [30].

Notably, Mendoza-Hernandez et al. (2025) reported that DENV-3 infection was not associated with classical warning signs such as hemorrhagic manifestations [30]. Instead, mortality was more accurately predicted by neurological involvement, systemic inflammation, or renal dysfunction [30]. In the present study, detailed clinical symptoms and laboratory parameters were unavailable, as only clinical classification reports were accessible. Consequently, we were unable to evaluate warning signs among DENV-2 and DENV-3 cases, particularly among fatal cases. These findings highlight the need to reassess current clinical criteria used to define dengue severity [27,30].

Several limitations should be acknowledged. The study population consisted exclusively of individuals with access to formal employment and health services, which may limit generalizability. Evidence from the Philippines suggests that socioeconomic determinants significantly influence dengue incidence. Provinces characterized by high population density, larger household size, higher poverty levels, increased per capita health expenditure, and lower latitude demonstrate a higher risk of dengue compared with regions at higher temperature ranges; such social determinants were not incorporated into the present analysis [14].

From a methodological perspective, some samples tested positive for the dengue virus but yielded negative serotyping results. This may reflect low viral load or RNA degradation, allowing detection of pan-DENV targets but not serotype-specific sequences [31]. Ariyaratne et al. reported the co-circulation of two DENV-3 genotypes during the 2023 outbreak in Sri Lanka, one of which evaded detection by CDC primers [32]. Furthermore, the higher mutation rate observed in DENV-3 may contribute to false-negative serotyping results due to undetected viral variants [33].

Dengue is a global problem, and research groups in different parts of the world have sought to identify the factors that most influence its prevalence. These range from models that use only climatological variables, like ours [34], to those that incorporate time series and even machine learning using AI [35].

Our model has some limitations, including the type of insecticide used and its application frequency in the region under study. However, it also has strengths; a prediction coefficient linked to the epidemiological week number, temperatures, and precipitation levels was established. We believe that one of the main contributions of this study is to highlight the importance of the combination of climatic conditions that increase the frequency of dengue cases. Based on these findings, this scenario predicts more cases of light rainfall, high minimum temperatures, and low maximum temperatures. This provides a starting point to reinforce prevention actions when these conditions occur, including population education, fumigation strategies, and others.

Finally, the predictive model explained only a limited proportion of dengue cases. Future analyses should incorporate additional determinants beyond climatic variables, including ecological, social, and demographic factors, to improve explanatory power [12,36]. Dengue outbreaks cannot be attributed solely to climate, vectors, or viral characteristics [37]. Factors such as urbanization [38], migration, tourism, fumigation, and community-based educational interventions aimed at preventive behaviors should also be systematically evaluated [39].

## 5. Conclusions

Climatic variables and temporal trends explain a modest proportion of dengue rates in northwestern Mexico, with the strongest effect observed in Jalisco. A serotype shift was observed across the years from D1 to D2 to D3. The D3 serotype was the most dominant during the 2024 outbreak and was not associated with dengue severity. Comprehensive dengue prevention and control strategies should integrate climatic surveillance with vector control, population mobility monitoring, and health system preparedness.

## Figures and Tables

**Table 1:** Distribution of diagnosed cases of dengue fever by year.

	Year										
	2017	2018	2019	2020	2021	2022	2023	2024	2025	Total	(%)
States in the northwestern area of the country											
Colima	0	1	1	76	29	45	296	2279	5	2732	(9.4)
Guanajuato	0	2	49	165	2	0	375	1438	3	2034	(7.0)
Jalisco	196	532	3337	1194	21	17	335	7933	70	13,635	(47.0)
Michoacán	0	0	285	842	59	25	314	2574	14	4113	(14.2)
Nayarit	7	25	285	398	23	5	97	2743	10	3593	(12.4)
Sinaloa	0	0	32	2	16	252	3	194	0	499	(1.7)
Sonora	0	0	41	249	816	214	39	1011	10	2380	(8.2)
Total	203	560	4030	2926	966	558	1459	18,172	112	28,986	(100)
DENV * serotype											
D1	139	349	854	417	82	45	357	531	0	2774	(9.6)
D2	3	189	2917	2233	745	425	298	541	0	7351	(25.4)
D3	0	0	1	1	48	58	520	12,962	48	13,638	(47.1)
D4	0	0	0	0	0	1	3	29	0	33	(0.1)
Not determined	61	22	258	275	91	29	281	4109	64	5190	(17.8)
Total	203	560	4030	2926	966	558	1459	18,172	112	28,986	(100)
Clinical classification											
Non-severe dengue	0	432	2739	2183	729	286	1027	11,755	54	19,205	(66.3)
Dengue with warning signs	0	102	1013	676	214	256	416	6151	57	8885	(30.7)
Severe dengue	0	10	263	65	22	16	14	265	1	656	(2.3)
Not clinically classified	203	16	15	2	1	0	2	1	0	240	(0.8)
Total	203	560	4030	2926	966	558	1459	18,172	112	28,986	(100)

* DENV: dengue virus.

**Table 2:** Distribution of diagnosed cases by geographic region (2017–2025).

	Colima	Guanajuato	Jalisco	Michoacán	Nayarit	Sinaloa	Sonora	Total
DENV * serotype								
D1	169	306	1319	619	176	29	156	2774
D2	203	99	4036	1226	563	217	1007	7351
D3	1676	1266	6240	1438	2076	201	741	13,638
D4	0	1	10	10	9	0	3	33
Not determined	684	362	2030	820	769	52	473	5190
Total	2732	2034	13,635	4113	3593	499	2380	28,986
Patient gender								
Male	1308	996	6070	1891	1619	198	1114	13,196
Female	1424	1038	7565	2222	1974	301	1266	15,790
Gender ratio (M:F)	1:1.09	1:1.04	1:1.25	1:1.18	1:1.22	1:1.52	1:1.14	1:1.20
Patient type								
Outpatient	1764	1681	10,003	3196	2152	299	1197	20,292
Hospitalized	965	351	3593	914	1438	200	1178	8639
Death	3	2	39	3	3	0	5	55
Clinical classification								
Non-severe dengue	1721	1505	9311	3280	1897	272	1219	19,205
Dengue with warning signs	977	501	3711	805	1634	218	1039	8885
Severe dengue	33	28	392	24	49	9	121	656
Not clinically classified	1	0	221	4	13	0	1	240

* DENV: dengue virus.

**Table 3:** DENV serotypes and clinical severity in northwestern Mexico from 2017 to 2025.

	Clinical Classification			
	Non-Severe Dengue	Dengue with Warning Signs	Severe Dengue	Unknown
**D1**	2102 (10.9)	462 (5.2)	55 (8.4)	155 (64.6)
**D2**	5187 (27.0)	1863 (21.0)	285 (43.4)	16 (6.7)
**D3**	9057 (47.2)	4386(49.4)	195 (29.7)	0 (0.0)
**D4**	28 (0.1)	4 (0.0)	1 (0.2)	0 (0.0)
Not determined	2831 (14.8)	2170 (24.4)	120 (18.3)	69 (28.7)
**Total**	19,205	8885	656	240

DENV: Dengue virus; frequency (percentage).

**Table 4:** Linear regression model.

Dependent Variable: Number of Dengue Cases							
	Colima	Guanajuato	Jalisco	Michoacán	Nayarit	Sinaloa	Sonora
Variable							
Week ^***α***^	0.126	0.300	1.410	0.388	0.227	0.466	0.096
Precipitation (mm)	-- *	−0.907	−5.373	-- *	-- *	−0.603	-- *
Minimum Temperature (°C)	3.437	2.358	17.553	2.892	3.451	-- *	-- *
Mean Temperature (°C)	−4.246	−2.407	−9.160	−3.359	−2.718	−0.516	−0.080
Maximum Temperature (°C)	1.958	-- *	−6.570	1.018	-- *	-- *	-- *
Constant	−22.871	14.459	143.080	−0.456	4.061	7.409	0.579
R^2^ Adjusted	0.226	0.226	0.217	0.256	0.111	0.151	0.049
*p*	<0.001	<0.001	<0.001	<0.001	<0.001	<0.001	<0.001

* Variable not included in the model by the software for the corresponding state; method: backward, pin = 0.05, pout = 0.01; ^***α***^ epidemiological week (52 weeks per year). All values presented for each variable are coefficients calculated by the statistical software using the raw data for each variable as the source.

## Data Availability

The data presented in this study is available upon request from the corresponding author due to privacy reasons.

## Abbreviations

The following abbreviations are used in this manuscript:

DENV	dengue virus
DWS	dengue with warning signs
HIS	Hydrological Information System
LAVIE-CIBO	Laboratorio de Apoyo a la Vigilancia e Investigación Epidemiológica del Centro de Investigación Biomédica de Occidente
NSD	non-severe dengue
RT-qPCR	quantitative real-time PCR
SD	severe dengue
VBD	vector-borne disease
